# Supplementary material for: Exotic alleles contribute to heat tolerance in wheat under field conditions
Source: Commun Biol. 2023 Jan 9;6:21. doi: 10.1038/s42003-022-04325-5 (PMC9829678; doi:10.1038/s42003-022-04325-5)
Supplement: Supplementary file 2 — Supplementary Information [file 42003_2022_4325_MOESM2_ESM.pdf]

Supplementary Figure 1

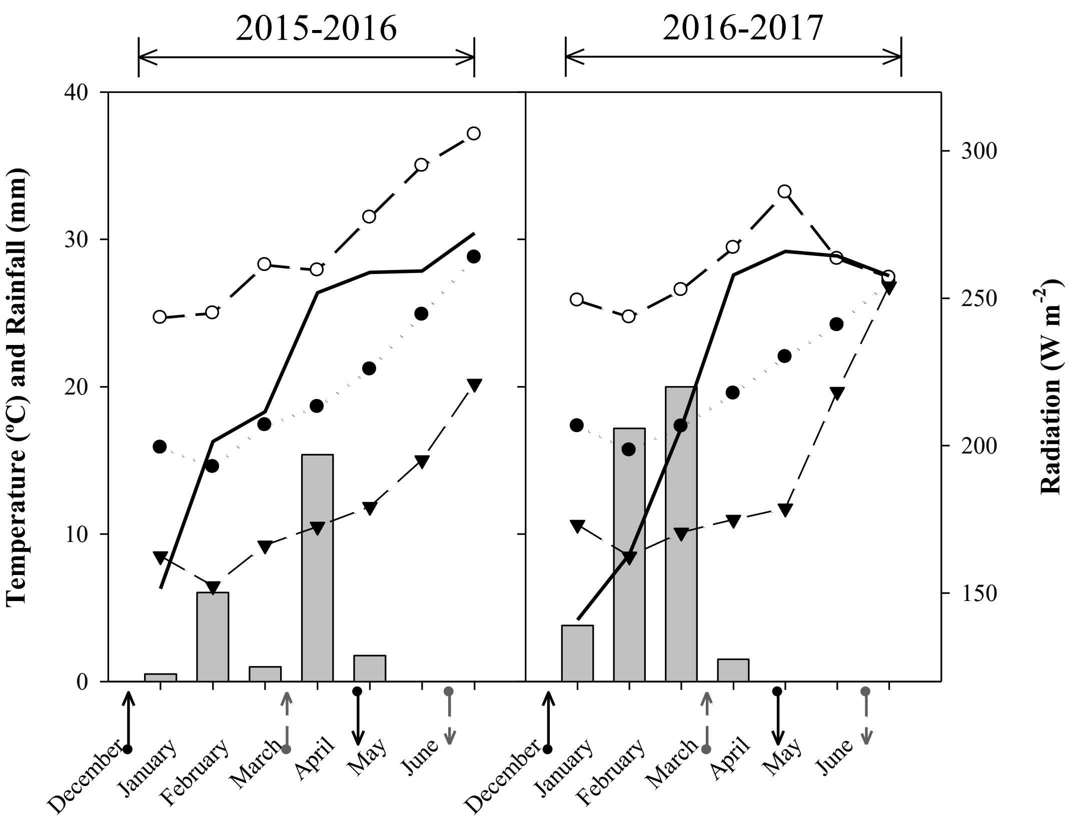

Supplementary Figure 1. Monthly accumulated rainfall (grey bars), average mean temperature (●), average maximum temperature (○), average minimum temperature (▼) and average monthly radiation (▬) registered during the experiments in the IWYP-HUB situated at CENEB experimental station situated in Ciudad Obregon Sonora, NW-Mexico. (↑) Represents emergence date for yield potential trials, (↓) represents harvest date for yield potential trials, (↑, broken line) represents emergence date for heat stressed trials, (↓, broken line) represents harvest date for heat stressed trials.

Supplementary Figure 2

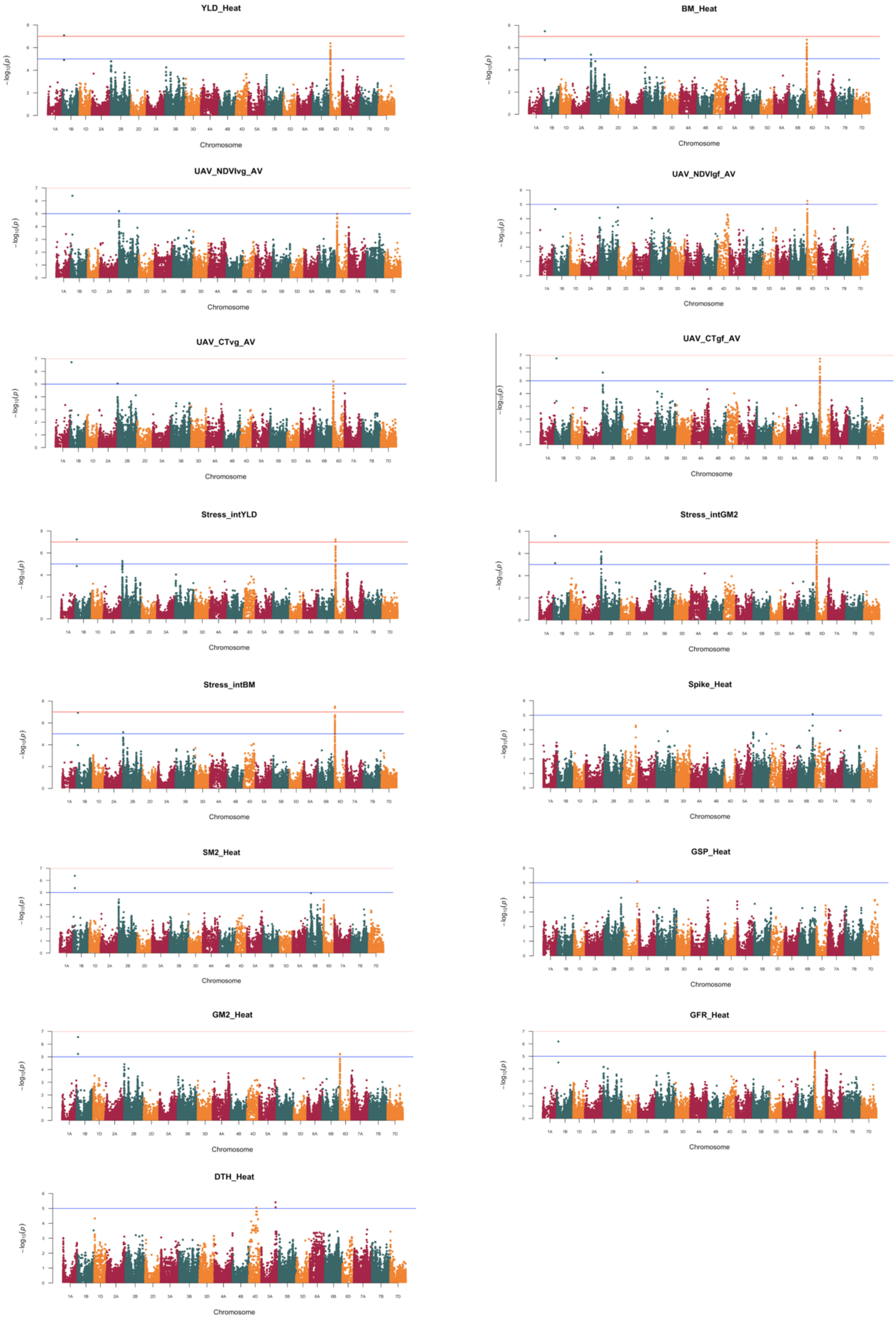

Supplementary Figure 2. GWAS results in HiBAP I based on BLUEs means obtained from the combined analysis from Ht16 and Ht17. The dotted horizontal line indicates threshold of significance.

Supplementary Figure 3

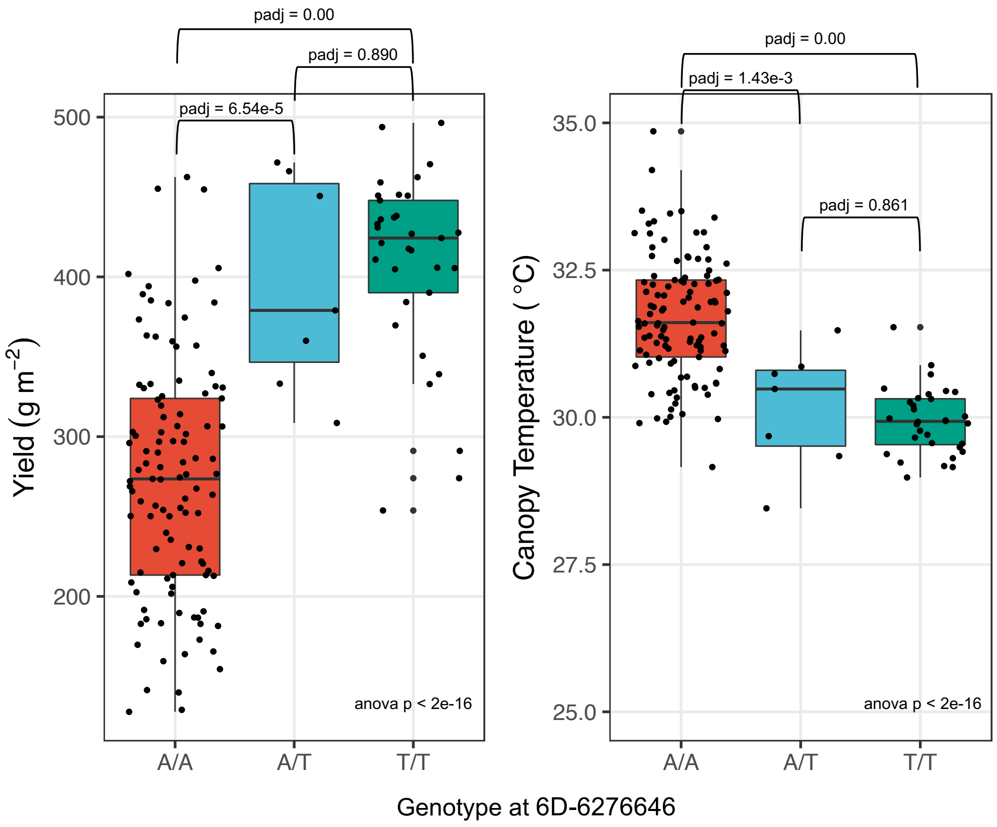

Supplementary Figure 3. Yield and vegetative canopy temperature under heat stressed conditions for lines with homozygous unfavourable allele (A/A), heterozygous for the favourable allele (A/T) and homozygous for the favourable allele (T/T). Black points indicate individual data points; this was done due to the small sample size of A/T. Significance was computed using a one-way ANOVA test (n=109, 7, and 32 biologically independently lines for A/A, A/T and T/T, respectively). Tukey's honest significance test was used to calculate adjusted p-values for each pairwise comparison.

Supplementary Figure 4

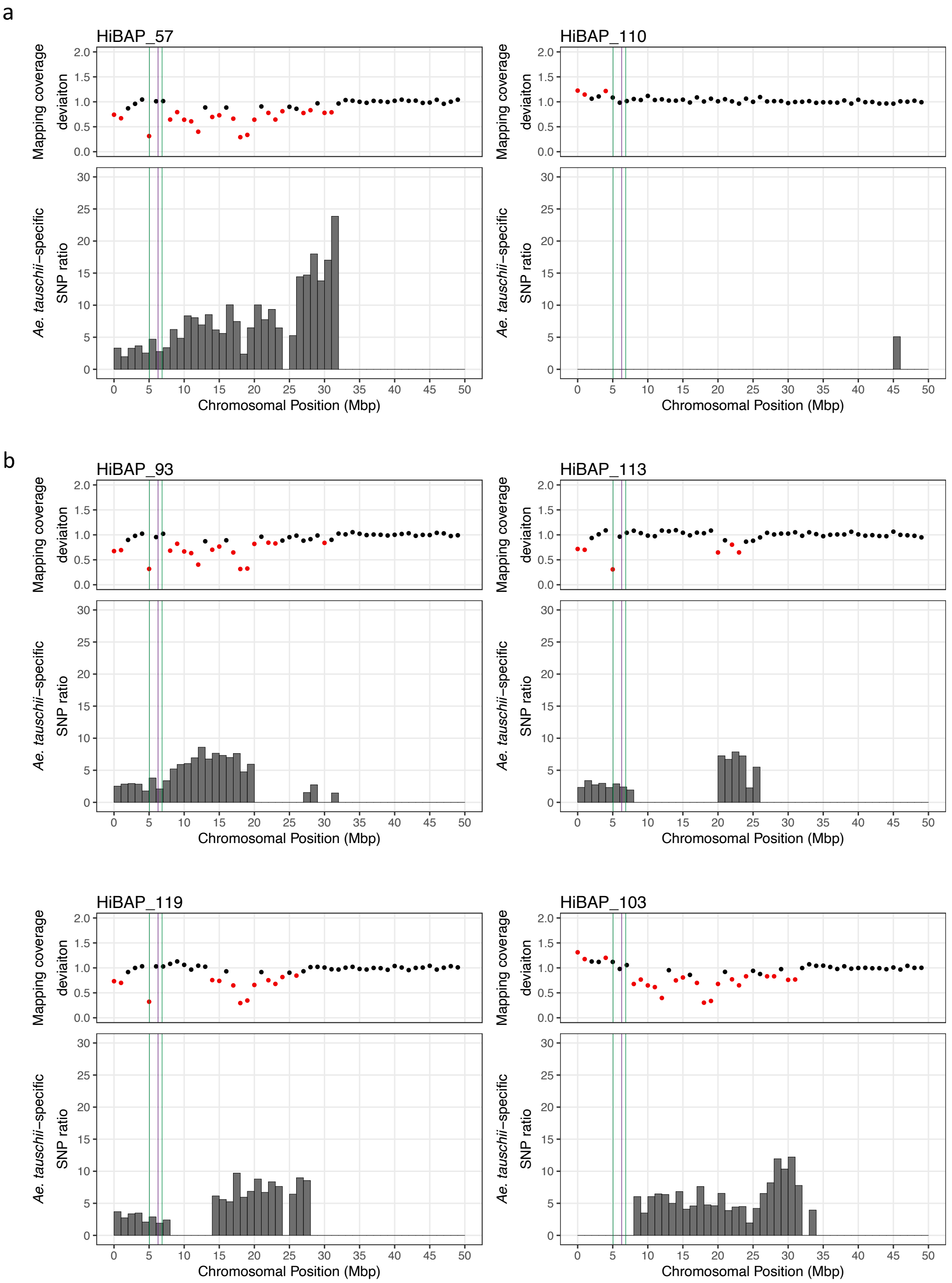

Supplementary Figure 4. *Ae. tauschii* introgressions within 6D:0-50Mbp in **a** Sokoll (HiBAP_57) and Weebil1 (HiBAP_110) and **b** four independent Sokoll/Weebil1 crosses. Mapping coverage deviation was computed between the HiBAP line and the median of the panel in 1Mbp windows. Red points are statistically significant outliers. *Ae. tauschii*-specific SNP ratio in each 1Mbp window was calculated by dividing the number of homozygous *Am. muticum*-specific SNPs in that window by mean number of homozgyous SNPs in that window across the panel. Green lines mark the borders of the region common to all lines with the T haplotype, corresponding to a 1.80Mbp region in CS and a 1.49Mbp in *Ae. Tauschii.* The purple line indicates the 6D MTA position.

Supplementary Table 1: Experimental details for each panel evaluated in the present study among different years at the InternationalWheat Yield Partneship HuB (IWYP-HUB) situated at CENEB in the Yaqui Valley, near Ciudad Obregon, Sonora, Mexico.

	Env	Cycle	Plot size	#rows	Reps	Emergence date	Harvest date	Pre-sowing irrigation	Tmax >35ºC‡	Irrigations					
										1	2	3	4	5	6
**HiBAP I**	YP	2015 — 2016	6.4 m^2	4	4†	07-Dec-15	13-May-16	28-Nov-15	4	07-Jan-16	05-Feb-16	27-Feb-16	02-Mar-16		
**(n =149 lines)**		2016 — 2017	6.4 m^2	2	4†	30-Nov-16	26-Apr-17	07-Nov-16	9	27-Dec-16	27-Jan-17	03-Mar-17			
	Heat	2016	1.6 m^2	3	2	03-Mar-16	13-Jun-16	08-Feb-16	25	18-Mar-16	01-Apr-16	15-Apr-16	29-Apr-16	14-May-16	27-May-16
		2017	1.6 m^2	3	2	08-Mar-17	20-Jun-17	07-Feb-17	14	18-Mar-17	02-Apr-17	18-Apr-17	30-Apr-17	14-May-17	27-May-17

†Only 2 replicates were measured for most of the traits

‡From Emergence until Harvest

Supplementary **Table 2: Summary statistics for physiological traits measured under heat stress or yield potential conditions over 2 years. Confidence intervals and p-values calculated using two-tailed t tests with no assumption of equal variance to compare Elite lines (N=83) with exotic-derived lines (N=66).**

Trait	Condition	Elite mean ± S.D	Exotic mean ± S.D	95% confidence interval	p-value
**YLD (g m^{-2})**	Heat stress	265±69.6	365±88.1	[-126,-73.6]	9.61e-12
**TGW(g)**	Heat stress	32.3±3.36	35.7±3.27	[-4.43,-2.28]	7.66e-09
**GM2(no. grains m^{-2})**	Heat stress	8120±1913	10246±2054	[-2776,-1476]	1.685e-09
**BM_PM (g m^{-2})**	Heat stress	565±138	786±169	[-272,-170]	3.38e-14
**HI**	Heat stress	0.465±0.0232	0.466±0.0229	[-0.0009151, 0.000588]	0.668
**Height (cm)**	Heat stress	61.6±4.55	67.2±6.03	[-7.34,-3.80]	7.69e-09
**YLD (g m^{-2})**	Yield potential	560±42.0	590±35.6	[-2.51,22.6]	0.116
**TGW(g)**	Yield potential	42.4±3.95	45.7±4.06	[-4.69,-2.07]	1.03e-06
**GM2(no. grains m^{-2})**	Yield potential	14228±1066	12986±1374	[835,1649]	1.78e-08
**BM_PM (g m^{-2})**	Yield potential	1331±84.4	1385±97.2	[-83.6,-23.7]	5.54e-04
**HI**	Yield potential	0.478±0.0242	0.456±0.0225	[0.0140,0.0291]	9.21e-08
**Height (cm)**	Yield potential	97.9±3.99	102±4.98	[-5.22,-2.24]	2.41e-06

Supplementary Table 3. Average, minimum, maximum values, broad sense heritability (H2) and ANOVA for the main traits evaluated in the heat experiments and percentage of loss in comparison with experiments under favourable conditions.

Traits	HiBAP I (2015-2016 & 2016-2017)							
	Heat stress							%Loss *vs.* YP
	Mean	Max	Min	H^2	*P*(Gen)	*P*(Year)	*P(GxY)*	
YLD (gm^{-2})	309	496	128	0.79	**0.000**	0.071	**0.000**	-48.1
TGW (g)	33.8	42.2	23.4	0.88	**0.000**	0.969	**0.000**	-22.9
GM2 ($\#m^{-2}$)	9052	14370	4271	0.78	**0.000**	0.051	**0.000**	-33.8
BM_PM (gm^{-2})	663	1052	279	0.8	**0.000**	0.076	**0.000**	-51.1
HI	0.47	0.51	0.35	0.45	**0.000**	0.074	**0.001**	-0.7
SM2 ($\#m^{-2}$)	255	460	130	0.71	**0.000**	0.090	**0.000**	-15.9
GSP (#)	35.0†	46.9	22.6	0.31	**0.005**	**0.004**	**0.000**	-27.7
GWSP (g)	1.2†	1.7	0.6	0.62	**0.000**	**0.013**	**0.000**	-44.1
Plant Height (cm)	64	80.4	46.8	0.78	**0.000**	0.146	0.339	-35.7
$SPKLSP^{-1}$ (#)	18.2	20.2	14.7	0.67	**0.000**	0.599	0.963	-8.2
Infertile $SPKLSP^{-1}$ (#)	1.4	2.7	0.6	0.44	**0.000**	0.775	0.591	26.8
Spike Length (cm)	9.9	11.6	6.4	0.88	**0.000**	0.153	1	-17.8
Awns Length (cm)	5.3	7	4	0.76	**0.000**	0.054	1	-17.1
Peduncle Length (cm)	21.2	29.9	14.9	0.76	**0.000**	0.146	**0.000**	-43.5
DTA/DTH (days)	53	62	44	0.88	**0.000**	**0.027**	**0.000**	-31.2
DTM (days)	80	90	72	0.87	**0.000**	0.105	**0.000**	-30.6
Grain filling (%)	34	44.8	25.9	0.66	**0.000**	0.638	**0.000**	1.6

†COV DTH used to adjust the meas as its effect was significant

YLD: grain yield, TGW: thousand grain weight, GM2: grains per m2, BM_PM: biomass at physiological maturity, HI: Harvest Index, SM2: spikes per m2, GSP: grains per spike, GWSP: grain weight per spike, SPKLSP: spikeletes per spike, DTA: days to anthesis, DTH: days to heading, DTM: days to maturity

Supplementary Table 4. Pearson's correlation tests between NDVI and Yield and between Canopy temperature and yield under either heat stressed or yield potential conditions and at either the vegetative or grain filling phenological stage.

Trait	Condition	Phenological stage	Line classification	Pearson's correlation coefficient (r)	95% confidence interval	p-value
**NDVI**	Heat stress	Vegetative	Elite	0.753	[0.641,0.833]	2.29e-16
**NDVI**	Heat stress	Vegetative	Exotic-derived	0.814	[0.712,0.882]	<2.2e-16
**NDVI**	Yield potential	Vegetative	Elite	-0.197	[-0.396,0.0197]	0.0745
**NDVI**	Yield potential	Vegetative	Exotic-derived	-0.187	[-0.410,0.0579]	0.133
**NDVI**	Heat stress	Grain Filling	Elite	0.444	[0.252,0.602]	2.64e-05
**NDVI**	Heat stress	Grain Filling	Exotic-derived	0.712	[0.568,0.814]	2.00e-11
**NDVI**	Yield potential	Grain Filling	Elite	-0.256	[-0.447,-0.0425]	0.0196
**NDVI**	Yield potential	Grain Filling	Exotic-derived	0.0943	[-0.151,0.329]	0.451
**CT**	Heat stress	Vegetative	Elite	-0.758	[-0.837,-0.648]	<2.2e-16
**CT**	Heat stress	Vegetative	Exotic-derived	-0.875	[-0.922,-0.804]	<2.2e-16
**CT**	Yield potential	Vegetative	Elite	-0.260	[-0.450,-0.0467]	0.0177
**CT**	Yield potential	Vegetative	Exotic-derived	0.00350	[-0.239,0.245]	0.978
**CT**	Heat stress	Grain Filling	Elite	-0.702	[-0.797,-0.573]	1.44e-13
**CT**	Heat stress	Grain Filling	Exotic-derived	-0.859	[-0.912,-0.779]	<2.2e-16
**CT**	Yield potential	Grain Filling	Elite	-0.206	[-0.403,0.0105]	0.0621
**CT**	Yield potential	Grain Filling	Exotic-derived	-0.265	[-0.476,-0.0243]	0.0317

Supplementary Table 5. Phenotypic correlations between NDVI and CT measured in the heat experiments at the vegetative stage (vg) or during grain filling (gf) and Stress Susceptibility Index calculated for yield (SSI_YLD) with main yield and yield components. Bold indicates significant at at least P<0.05

Traits	NDVIvg_AV	NDVIgf_AV	CTvg_AV	CTgf_AV	SSI_YLD
YLD (gm^{-2})	**0.849**	**0.700**	**-0.871**	**-0.851**	**-0.976**
TGW (g)	**0.591**	**0.472**	**-0.594**	**-0.574**	**-0.586**
GM2 (#m^{-2})	**0.795**	**0.659**	**-0.816**	**-0.801**	**-0.928**
BM_PM (gm^{-2})	**0.874**	**0.746**	**-0.895**	**-0.879**	**-0.962**
HI	**0.185**	0.057	**-0.195**	**-0.174**	**-0.369**
SM2 (#m^{-2})	**0.670**	**0.588**	**-0.680**	**-0.678**	**-0.829**
GSP (#)	**0.472**	**0.349**	**-0.500**	**-0.475**	**-0.455**
GWSP (g)	**0.665**	**0.518**	**-0.687**	**-0.658**	**-0.665**
Plant Height (cm)	**0.722**	**0.612**	**-0.727**	**-0.734**	**-0.680**
SPKLSP^{-1} (#)	**0.405**	**0.384**	**-0.392**	**-0.375**	**-0.219**
Infertile SPKLSP^{-1} (#)	-0.010	0.063	-0.010	-0.015	0.046
Spike Length (cm)	**0.407**	**0.311**	**-0.398**	**-0.371**	**-0.219**
Awns Length (cm)	**0.171**	0.119	-0.153	**-0.186**	-0.093
Peduncle Length (cm)	**0.536**	**0.438**	**-0.506**	**-0.522**	**-0.486**
DTA/DTH (days)	**0.270**	**0.504**	**-0.211**	**-0.268**	0.009
DTM (days)	**0.428**	**0.680**	**-0.353**	**-0.428**	**-0.181**
Grain filling (%)	0.045	-0.055	-0.060	-0.053	**-0.232**
NDVIvg_AV	-	**0.879**	**-0.954**	**-0.945**	**-0.850**
NDVIgf_AV	**0.879**	-	**-0.847**	**-0.905**	**-0.719**
CTvg_AV	**-0.954**	**-0.847**	-	**0.951**	**0.863**
CTgf_AV	**-0.945**	**-0.905**	**0.951**	-	**0.856**

Supplementary Table 6. Summary of Marker-Trait Associations (MTAs) for different physiological traits and the chromosomes where they were identified, position, P-value and interval.

Trait	Chromosome	MTA ID	Position	P-value	Interval
**Yield Traits**					
**YLD**	chr1B	chr1B-63398861	63398861	8.32E-08	0.6-10Mbp
	chr6D	chr6D-6276646	6276646	4.16E-07	3-7.5Mbp
**BM**	chr1B	chr1B-63398861	63398861	3.4E-08	0.6-10Mbp
	chr2B	chr2B-820002	820002	0.00000434	1Mbp
	chr6D	chr6D-6276646	6276646	1.94E-07	3-7.5Mbp
**GFR**	chr1B	chr1B-63398861	63398861	6.55E-07	0.6-10Mbp
	chr6D	chr6D-6276646	6276646	0.00000439	3-7.5Mbp
**GM2**	chr1B	chr1B-63398861	63398861	6.4806E-06	0.6-10Mbp
	chr6D	chr6D-6276646	6276646	2.8069E-07	3-7.5Mbp
**SM2**	chr1B	chr1B-63398861	63398861	4.1647E-07	0.6-10Mbp
**Stress Tolerance Index**					
**Stress_intYLD**	chr1B	chr1B-63398861	63398861	5.9082E-08	0.6-10Mbp
	chr2B	chr2B-820002	820002	5.4266E-06	1Mbp
	chr6D	chr6D-6276646	6276646	5.9158E-08	3-7.5Mbp
**Stress_intBM**	chr1B	chr1B-63398861	63398861	3.1404E-08	0.6-10Mbp
	chr2B	chr2B-820002	820002	7.1692E-06	1Mbp
	chr6D	chr6D-6276646	6276646	3.1404E-08	3-7.5Mbp
**Stress_intGM2**	chr1B	chr1B-63398861	63398861	2.6569E-08	0.6-10Mbp
	chr2B	chr2B-820002	820002	7.1074E-07	1Mbp
	chr6D	chr6D-6276646	6276646	6.6123E-08	3-7.5Mbp
**UAV measurements**					
**UAV_CTvg_AV**	chr1B	chr1B-63398861	63398861	1.8924E-07	0.6-10Mbp
	chr2B	chr2B-820002	820002	9.0008E-06	1Mbp
	chr6D	chr6D-6276646	6276646	6.1095E-06	3-7.5Mbp
**UAV_CTgf_AV**	chr1B	chr1B-63398861	63398861	1.7673E-07	0.6-10Mbp
	chr2B	chr2B-820002	820002	2.2766E-06	1Mbp
	chr6D	chr6D-6276646	6276646	1.8452E-07	3-7.5Mbp
**UAV_NDVIvg_AV**	chr1B	chr1B-63398861	63398861	4.0814E-07	0.6-10Mbp
	chr2B	chr2B-820002	820002	6.4281E-06	1Mbp
**UAV_NDVIgf_AV**	chr6D	chr6D-6276646	6276646	5.7295E-06	3-7.5Mbp
**Phenology**					
**DTH_Heat**	chr4D	chr4D-299997620	299997620	0.00000875	1Mbp
	chr5A	chr5A-581257473	581257473	0.00000387	580-568Mbp

Supplementary Table 7. Yield and canopy temperature of the different allele combinations at the MTAs chr6D-6276646, 1B chr1B-63398861, and 2B chr2B-820002, where the combination of favourable alleles is T+C+C and the combination of unfavourable alleles is A+A+G. Significance of allele combinations was computed using a one-way ANOVA test and post-hoc test using Tukey's honest significance test

			Mean ± S.D			Tukey's HSD p-values		
**Trait**	**Condition**	**Anova p-value**	**A+A+G**	**A+C+C**	**T+C+C**	A+C+C-A+A+G	T+C+C-A+A+G	T+C+C-A+C+C
**Yield**	Heat stress	<2e-16	263±67.3 g m^{-2}	327±79.5 g m^{-2}	411±59.8 g m^{-2}	2.58e-3	0	3.18e-4
**Yield**	Yield Potential	0.238	600±44.8 g m^{-2}	591±28.5 g m^{-2}	587±27.7 g m^{-2}	0.681	0.242	0.937
**CT**	Heat stress	<2e-16	31.8±0.987 °C	31.0±0.963 °C	29.9±0.556 °C	5.97e-3	0	2.21e-4
**CT**	Yield Potential	0.210	26.0±0.255 °C	26.0±0.318 °C	25.9±0.243 °C	0.676	0.211	0.921

Supplementary Table 8: Summary of the UAV platforms, together with the cameras and the achieved used to evaluate HiBAP I under yield potential and Heat stress conditions.

Multispectral (NDVI)				
Cycle	Platform	Camera	Flight altitude	GSD (cm)
2015-2016	Falcon 8	ADC Lite (Tetracam, USA)	70 m	2.7
2016-2017	Matrice 100	RedEdge (MicaSense)	30 m	2
Thermal (CT)				
Cycle	Platform	Camera	Flight altitude	GSD (cm)
2015-2016	Falcon 8	TAU 640 (FLIR, USA)	80 m	5.4
2016-2017	Matrice 100	Zenmuse XT (DJI, China)	40 m	5.2

Supplementary Note 1. Evidence linking proposed candidate genes to heat tolerance

We found two type-B two-component response regulator genes, with members of this family acting as transcription factors in the cytokinin signalling pathway (1). They have been linked to photoperiod stress protection via root-derived cytokinins (2) and negative regulation of drought response (3) via root cytokinin pathways (4); the resulting elevated levels of cytokinin have been linked to heat stress tolerance (5). We also found a MIKC-type MADS-box transcription factor orthologous to the *Arabidopsis* gene *SOC1*, overexpression of which leads to chloroplast biogenesis, elevated photosynthesis, and tolerance to prolonged heat stress (6). Finally, we identified a novel MAPK gene. MAPKs play important roles in regulating responses to abiotic stress. They have been linked to oxidative stress tolerance under heat stress in wheat (7) and are involved in the heat stress response in *Arabidopsis*, maize and rice (8). The novelty of this gene and the presence-absence variation between *Ae. tauschii* accessions suggests this gene is recently evolved and possibly involved in environmental adaptation. The gene *DREB1A* was identified within the chr2B-820002 interval; this gene is part of a family of plant-specific transcription factors that bind DRE/CRT elements in the response to abiotic stresses. Overexpression of *DREB1* in wheat led to drought tolerance and increased photosynthetic efficiency (9) and *DREB2* overexpression lines displayed cold and heat tolerance (10). The 2B interval also contained *STEROL GLUCOSYLTRANSFERASE* (*SGT*) which has been identified to affect heat stability in both knockout and overexpression studies in *Arabidopsis* (11, 12).

Supplementary References

1. R. D. Argyros, *et al.*, Type B Response Regulators of Arabidopsis Play Key Roles in Cytokinin Signaling and Plant Development. *Plant Cell* **20**, 2102 (2008).
2. M. Frank, A. Cortleven, O. Novák, T. Schmülling, Root-derived trans-zeatin cytokinin protects Arabidopsis plants against photoperiod stress. *Plant. Cell Environ.* **43**, 2637–2649 (2020).
3. K. H. Nguyen, *et al.*, Arabidopsis type B cytokinin response regulators ARR1, ARR10, and ARR12 negatively regulate plant responses to drought. *Proc. Natl. Acad. Sci. U. S. A.* **113**, 3090–3095 (2016).
4. X. Huang, *et al.*, The Antagonistic Action of Abscisic Acid and Cytokinin Signaling Mediates Drought Stress Response in Arabidopsis. *Mol. Plant* **11**, 970–982 (2018).
5. Y. Liu, M. Zhang, Z. Meng, B. Wang, M. Chen, Research Progress on the Roles of Cytokinin in Plant Response to Stress. *Int. J. Mol. Sci.* **21**, 6574 (2020).
6. G. Ning, *et al.*, Genetic manipulation of Soc1-like genes promotes photosynthesis in flowers and leaves and enhances plant tolerance to high temperature. *Plant Biotechnol. J.* **19**, 8–10 (2021).
7. R. R. Kumar, *et al.*, Characterizing the putative mitogen-activated protein kinase (MAPK) and their protective role in oxidative stress tolerance and carbon assimilation in wheat under terminal heat stress. *Biotechnol. Reports* **29**, e00597 (2021).
8. S. Mo, *et al.*, Mitogen-activated protein kinase action in plant response to high-temperature stress: a mini review. *Protoplasma* **258**, 477–482 (2021).
9. Y. Zhou, *et al.*, Overexpression of soybean DREB1 enhances drought stress tolerance of transgenic wheat in the field. *J. Exp. Bot.* **71**, 1842–1857 (2020).
10. S. ji Lee, *et al.*, DREB2C Interacts with ABF2, a bZIP Protein Regulating Abscisic Acid-Responsive Gene Expression, and Its Overexpression Affects Abscisic Acid Sensitivity. *Plant Physiol.* **153**, 716–727 (2010).
11. M. K. Mishra, *et al.*, Overexpression of WsSGTL1 Gene of Withania somnifera Enhances Salt Tolerance, Heat Tolerance and Cold Acclimation Ability in Transgenic Arabidopsis Plants. *PLoS One* **8** (2013).
12. P. Misra, G. Singh, M. K. Mishra, V. Pandey, S. Saema, Functional Analysis and the Role of Members of SGT Gene Family of Withania somnifera. *Transgenes. Second. Metab.*, 1–14 (2016).
