# Supplementary material for: Exotic alleles contribute to heat tolerance in wheat under field conditions
Source: Commun Biol. 2023 Jan 9;6:21. doi: 10.1038/s42003-022-04325-5 (PMC9829678; doi:10.1038/s42003-022-04325-5)
Supplement: Supplementary file 3 — Description of Additional Supplementary Data [file 42003_2022_4325_MOESM3_ESM.docx]

**Description of Additional Supplementary Files**

**File name:** Supplementary Data 1

**Description:** Metadata of HIBAP I lines used in this study

**File name:** Supplementary Data 2

**Description:** Genes present within the core introgressed region in each of five Ae. tauschii accessions

**File name:** Supplementary Data 3

**Description:** Source data used to produce the main figure
