# Supplementary material for: Exotic alleles contribute to heat tolerance in wheat under field conditions
Source: Commun Biol. 2023 Jan 9;6:21. doi: 10.1038/s42003-022-04325-5 (PMC9829678; doi:10.1038/s42003-022-04325-5)
Supplement: Supplementary file 7 — Reporting Summary-New [file 42003_2022_4325_MOESM7_ESM.pdf]

nature portfolio

Corresponding author(s): Anthony Hall and Matthew Reynolds

Last updated by author(s): Nov 22, 2022

Reporting Summary

Nature Portfolio wishes to improve the reproducibility of the work that we publish. This form provides structure for consistency and transparency in reporting. For further information on Nature Portfolio policies, see our Editorial Policies and the Editorial Policy Checklist.

Statistics

For all statistical analyses, confirm that the following items are present in the figure legend, table legend, main text, or Methods section.

n/a	Confirmed	
☐	☒	The exact sample size (*n*) for each experimental group/condition, given as a discrete number and unit of measurement
☐	☒	A statement on whether measurements were taken from distinct samples or whether the same sample was measured repeatedly
☐	☒	The statistical test(s) used AND whether they are one- or two-sided<br>*Only common tests should be described solely by name; describe more complex techniques in the Methods section.*
☐	☒	A description of all covariates tested
☐	☒	A description of any assumptions or corrections, such as tests of normality and adjustment for multiple comparisons
☐	☒	A full description of the statistical parameters including central tendency (e.g. means) or other basic estimates (e.g. regression coefficient) AND variation (e.g. standard deviation) or associated estimates of uncertainty (e.g. confidence intervals)
☐	☒	For null hypothesis testing, the test statistic (e.g. F, t, r) with confidence intervals, effect sizes, degrees of freedom and P value noted<br>*Give P values as exact values whenever suitable.*
☒	☐	For Bayesian analysis, information on the choice of priors and Markov chain Monte Carlo settings
☒	☐	For hierarchical and complex designs, identification of the appropriate level for tests and full reporting of outcomes
☐	☒	Estimates of effect sizes (e.g. Cohen's *d*, Pearson's *r*), indicating how they were calculated

Our web collection on statistics for biologists contains articles on many of the points above.

Software and code

Policy information about availability of computer code

Data collection	Pix4D<br>R package 'raster'
Data analysis	METAR<br>BWA v0.7.13<br>Samtools v1.4<br>Picard tools v2.1.1<br>bcftools v1.3.1<br>GATK v3.5.0<br>Beagle v5.0<br>STRUCTURE v2.3.4<br>CLUMPP v1.1.2<br>GAPIT v3.0<br>bedtools v2.28.0<br>R package 'outliers' v0.15<br>R package 'pafr' v0.0.2<br>blast+ v2.7.1<br>minimap2.2.7<br>HMMER v3.3<br>HISAT2 v2.0.4<br>EMBOSS v6.6.0

For manuscripts utilizing custom algorithms or software that are central to the research but not yet described in published literature, software must be made available to editors and reviewers. We strongly encourage code deposition in a community repository (e.g. GitHub). See the Nature Portfolio guidelines for submitting code & software for further information.

Data

Policy information about availability of data

All manuscripts must include a data availability statement. This statement should provide the following information, where applicable:

- Accession codes, unique identifiers, or web links for publicly available datasets
- A description of any restrictions on data availability
- For clinical datasets or third party data, please ensure that the statement adheres to our policy

Publicly available sequencing data used in this study is available at the European Nucleotide Archive (ENA): HiBAP I enrichment capture sequencing data - PRJEB38874; Th. ponticum – SRR13484812; S. vavilovii: ERR505040, ERR505041, ERR505042; S. cereale accession Lo90: ERR504990, ERR504991, ERR504992; S. cereale accession Lo176: ERR505005, ERR505006, ERR505007; S. cereale accession Lo282: ERR505015, ERR505016, ERR505017; S. cereale accession Lo351: ERR505035, ERR505036, ERR505037; Ae. Tauschii accession XJ65: SRR13961980; Y173: SRR13962062; SX60: SRR13962012; AY29: SRR13961834; KU2832: SRR13961928; Y215: SRR13962048; Weebil1: PRJEB35709; Norin61: PRJNA492239; Pavon76: https://opendata.earlham.ac.uk/wheat/under_license/toronto/Hall_2021-10-08_wheatxmuticum/PIP-2495/200812_A00478_0126_AHN5W3DRXX/A10948_1_1/; Ae. tauschii RNAseq data: PRJEB23317; T. aestivum cv. Chinese Spring RNAseq data: Root - SRP133837; SRR6799264; SRR6799265; Leaf - SRR6799258; SRR6799259; SRR6799260; Spike - SRR6802608; SRR6802609; SRR6802610; SRR6802611.
VCF and hapmap genotype files for HiBAP I are available at: https://opendata.earlham.ac.uk/wheat/under_license/toronto/Hall_2022-04-08_HiBAP_genotyping/
Phenotypic data presented in this paper for the HIBAP I panel evaluated under yield potential and heat stressed environments can be found in the Dataverse CIMMYT Research Data Repository at https://data.cimmyt.org/dataset.xhtml?persistentId=hdl:11529/10548643

Code and source data needed to reproduce the main figures can be found at the github repository: https://github.com/benedictcoombes/Exotic_alleles_contribute_to_heat_tolerance_in_wheat_under_field_conditions

Human research participants

Policy information about studies involving human research participants and Sex and Gender in Research.

Reporting on sex and gender	N/A
Population characteristics	N/A
Recruitment	N/A
Ethics oversight	N/A

Note that full information on the approval of the study protocol must also be provided in the manuscript.

Field-specific reporting

Please select the one below that is the best fit for your research. If you are not sure, read the appropriate sections before making your selection.

☒ Life sciences ☐ Behavioural & social sciences ☐ Ecological, evolutionary & environmental sciences

For a reference copy of the document with all sections, see nature.com/documents/nr-reporting-summary-flat.pdf

Life sciences study design

All studies must disclose on these points even when the disclosure is negative.

Sample size	149 wheat lines, comprising 83 Elite lines and 66 exotic-derived lines (15 alien introgression lines, 11 Mexican and other origin landrace derived lines, 26 synthetic-derived lines and 14 synthetic+landrace derived lines). The size of the panel was considered based on previous publications aiming to have enough genetic resolution to identify interesting marker association, while at the same time being able to conduct all the phenotypic measurements across replicates, environments and years. The biomass and yield components are very time-consuming and not easy to manage in a breeding program with limited resources. This is why this population size was considered appropriate to have a complete phenotypic dataset . The suitability of the panel size has been confirmed by Molero et al., 2019 (PBJ, 17:1276) and Joynson et al., 2021 (PBJ, 19:6403).
Data exclusions	The original HiBAP I panel consisted on 150 lines. However, one line was a durum wheat (Triticum turgidum) while the rest of the panel was composed by bread wheats (Triticum aestivum). Therefore, we decided to remove it from the paper as we did in Joynson et al., 2021. PBJ 19 (6403). Any other data was excluded from the analysis.

Replication	Phenotype data was collected over 2 years (2 different growing seasons)<br>Yield potential experiments consisted of four replicates in raised beds (2 beds per plot each 0.8 m wide) with four (YP16) and two (YP17) rows per bed (0.1 m and 0.24 m between rows respectively) and 4 m long. For heat stressed experiments, two replicates were evaluated for HiBAP I in 2m×0.8m plots with three rows per bed. Overall heritabilities for all the traits were medium to high for yield potential (Molero et al., 2019 PBJ) and heat (Supplementary Table S3). This confirms that the data is consistent and repeatable enough to have solid results.
Randomization	Experimental design for both environments was an alpha-lattice. Days to anthesis/heading was used as covariate when its effect was significant at $p<0.05$.
Blinding	N/A

Reporting for specific materials, systems and methods

We require information from authors about some types of materials, experimental systems and methods used in many studies. Here, indicate whether each material, system or method listed is relevant to your study. If you are not sure if a list item applies to your research, read the appropriate section before selecting a response.

Materials & experimental systems

n/a	Involved in the study
☒	☐ Antibodies
☒	☐ Eukaryotic cell lines
☒	☐ Palaeontology and archaeology
☒	☐ Animals and other organisms
☒	☐ Clinical data
☒	☐ Dual use research of concern

Methods

n/a	Involved in the study
☒	☐ ChIP-seq
☒	☐ Flow cytometry
☒	☐ MRI-based neuroimaging
